# A Novel Patient‐Specific Landmark‐Guided Approach for Intramuscular Botulinum Neurotoxin Injections Into the Rotator Cuff: A Cadaveric Study

**DOI:** 10.1002/ca.70068

**Published:** 2025-12-23

**Authors:** Dave Osinachukwu Duru, David Kwan Ryung Lee, Gavin E. Jarvis, Niel Kang, Salma Chaudhury, Cecilia Brassett

**Affiliations:** ^1^ Human Anatomy Centre, Department of Physiology, Development and Neuroscience University of Cambridge Cambridge UK; ^2^ Gonville & Caius College, University of Cambridge Cambridge UK; ^3^ School of Medicine, University of Sunderland Sunderland UK; ^4^ Department of Trauma and Orthopaedics Cambridge University Hospitals NHS Foundation Trust Cambridge UK

**Keywords:** botulinum neurotoxin (BoNT), cadaveric, injection, landmark‐guided, motor points, myofascial pain, patient‐specific, rotator cuff, suprascapular nerve, variation

## Abstract

Myofascial pain syndrome (MFPS) causes chronic shoulder pain. Supraspinatus and infraspinatus, rotator cuff muscles innervated by the suprascapular nerve, are commonly affected. Intramuscular botulinum neurotoxin (BoNT) injections near motor points (i.e.*,* visible nerve branch entry sites used as a proxy for motor endplates) are an effective treatment for such pain. However, current techniques limit accessibility. This study aimed to develop a patient‐specific, landmark‐guided technique for BoNT delivery into supraspinatus and infraspinatus using scapular dimensions. Ten pairs of cadaveric shoulders (*n* = 20) were dissected to identify supraspinatus and infraspinatus motor points. Distances from scapular landmarks to these motor points were measured in two axes. These distances were correlated with scapular dimensions (height, spine length, width) using linear regression. Patient‐specific predictive formulae were derived. For validation, landmark‐guided methylene blue dye injections were performed on four additional shoulders using calculated coordinates. For supraspinatus, motor points were predicted using deltoid tubercle and root of the scapular spine (*r* = 0.58–0.64, *p* = 0.0016–0.021). For infraspinatus, root of the scapular spine and lateral acromion were used (*r* = 0.46–0.60, *p* = 0.0054–0.0500). In all validation specimens, injected dye accurately reached the motor points. This study provides a validated, patient‐specific, landmark‐guided technique for BoNT delivery into the rotator cuff, offering an approach for accessible analgesia.

## 1 Introduction

Shoulder pain is prevalent, afflicting about 50% of the world's population each year (Brox 2003). While its etiology is multifactorial, a common cause is myofascial pain (Hains et al. 2010). Myofascial pain is characterized by hyperirritable trigger points, tender taut bands of skeletal muscle, commonly located in the back, neck, and shoulder (Epstein et al. 2018; Gilchrist and Pokorná 2021).

Within the shoulder, myofascial trigger points (MTrPs) often involve the rotator cuff muscles (Perez‐Palomares et al. 2009). In particular, 60%–68% of myofascial shoulder pain cases involve supraspinatus, with 78% involving infraspinatus (Bron et al. 2011; Villafañe et al. 2019). Electrophysiological analyses suggest MTrPs tend to localize near motor points (Chu 1995; Simons et al. 2002). In this study, we define motor points as the visible entry sites of suprascapular nerve branches into the muscle belly, which serve as a practical anatomical proxy for underlying motor endplates (Harrison et al. 2007). Within the rotator cuff, MTrPs cause significant pain, limit range of motion, and diminish quality of life (Villafañe et al. 2019).

Myofascial pain can be managed by physiotherapy, topical vapocoolants, or local injections of corticosteroids, lidocaine, or botulinum neurotoxin (BoNT) (Cheshire et al. 1994; Shin et al. 2014; Xie et al. 2015; Kang et al. 2019).

BoNT inhibits acetylcholine release at the neuromuscular junction, producing prolonged muscle relaxation to relieve myofascial pain (Cheshire et al. 1994; De Andrés et al. 2003). Targeting BoNT injections closer to motor points improves efficacy (Miguel and Cirera 2021); however, this remains technically challenging due to extensive variation in motor point topography between patients (Lee et al. 2022, 2023). This variation also underscores a need for personalized approaches to injection targeting.

Current BoNT injection protocols rely on ultrasound or electromyography guidance (Evans and Porter 2015; Tan and Jia 2021). While effective, these techniques are not universally available and require specialist training. This highlights a clinical use for an anatomically validated, landmark‐guided injection approach.

This cadaveric study presents image‐free, patient‐specific approaches for landmark‐guided BoNT injection into supraspinatus and infraspinatus, targeting motor points. These approaches, informed by scapular dimensions, were validated through blind injection of methylene blue.

## 2 Materials and Methods

### 2.1 Specimen Preparation

Ten pairs of cadaveric shoulder specimens (*n* = 20) were provided by the Human Dissection Room, Anatomy Building, Department of Physiology, Development and Neuroscience, University of Cambridge, UK. The donors (5 male, 5 female; mean (SD) age = 80.8 (8.9) years) had provided consent for anatomical research prior to decease in compliance with the Human Tissue Act (2004). The donors were preserved by cannulation of the common carotid or femoral artery and injection under pressure of a solution containing 38% ethanol, 1.5% methanol, 4.2% formaldehyde, and 56.3% distilled water.

### 2.2 Defining Scapular Landmarks

The shoulder specimens were dissected to reveal six bony scapular landmarks: (1) superior angle; (2) inferior angle; (3) root of the scapular spine; (4) deltoid tubercle; (5) lateral acromion (defined as the mid‐aspect of the lateral edge of the acromion in line with the axis of the scapular spine); and (6) acromioclavicular (AC) joint (Figure 1). These landmarks were selected for their consistent bony morphology and ease of surface palpation.

**FIGURE 1:**
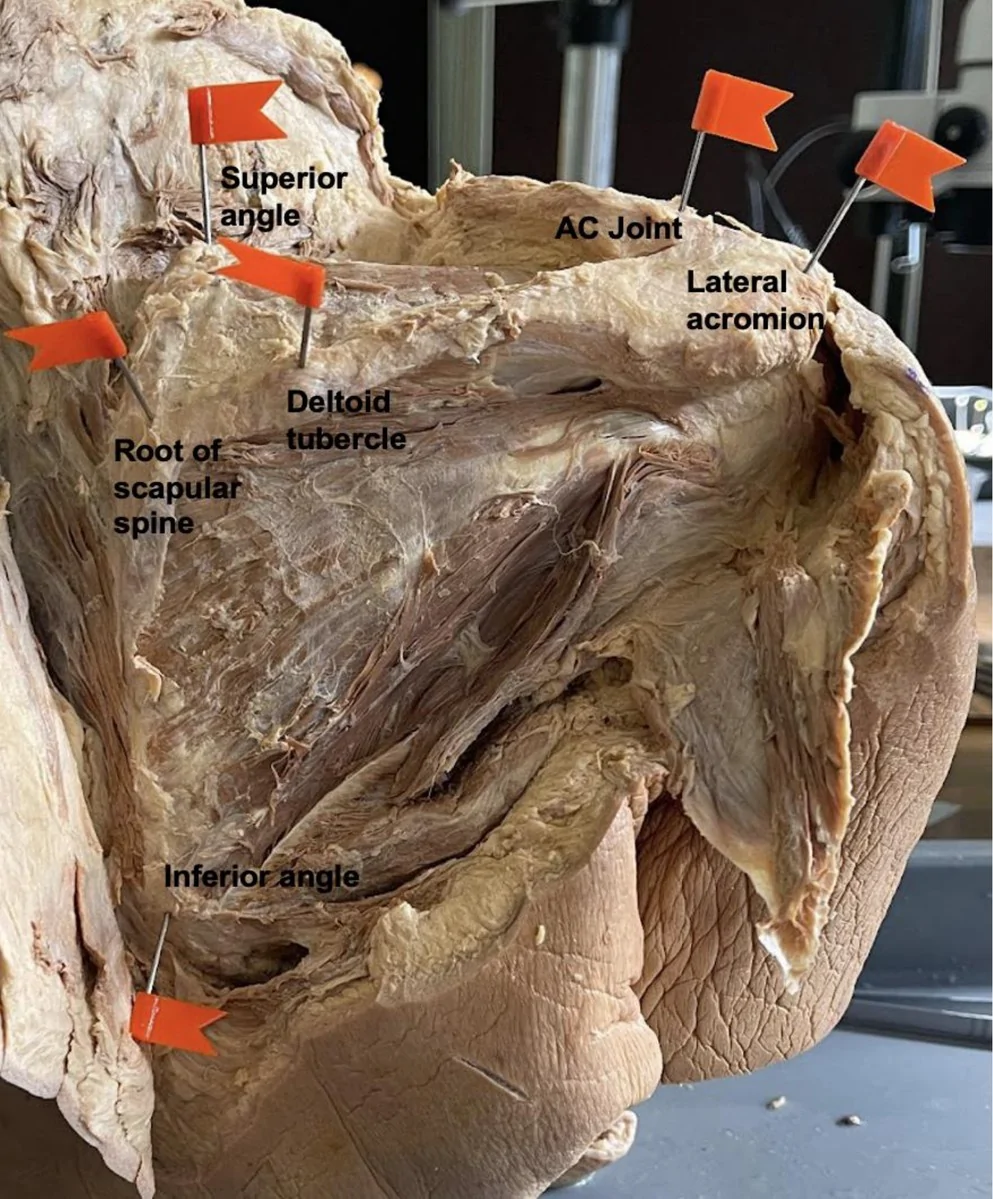
Bony scapular landmarks. Six landmarks of interest, used to guide injections into supraspinatus and/or infraspinatus. [Posterior view of right shoulder].

### 2.3 Measuring Scapular Dimensions

Scapular dimensions were measured, including scapular height, spine length, and width (Figure 2).

**FIGURE 2:**
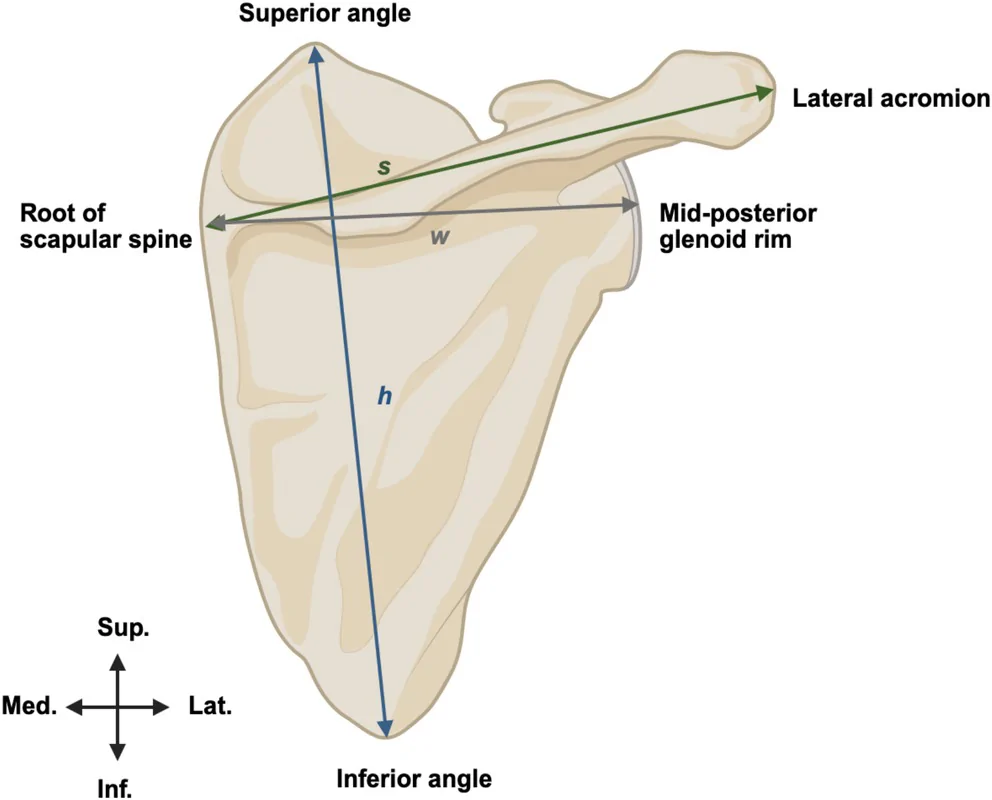
Measurement of scapular dimensions. Schematic figure of the posterior view of a right scapula. Height (h) = distance from the superior angle to the inferior angle. Spine length (s) = distance from the root of the scapular spine to the lateral acromion. Width (w) = distance from the root of the scapular spine to the mid‐posterior glenoid rim. Key: Inf., inferior; Lat., lateral; Med., medial; Sup., superior. [Figure made with BioRender].

### 2.4 Defining Key Points of Interest

Specimens were further dissected to visualize suprascapular nerve motor branch insertions into supraspinatus and infraspinatus. The muscles were reflected, and pins were inserted perpendicular to the underlying scapular bone surface to mark motor branch entry. The muscles were then laid into their in situ positions, as the nerve enters the muscle belly close to the bone (Figure 3).

**FIGURE 3:**
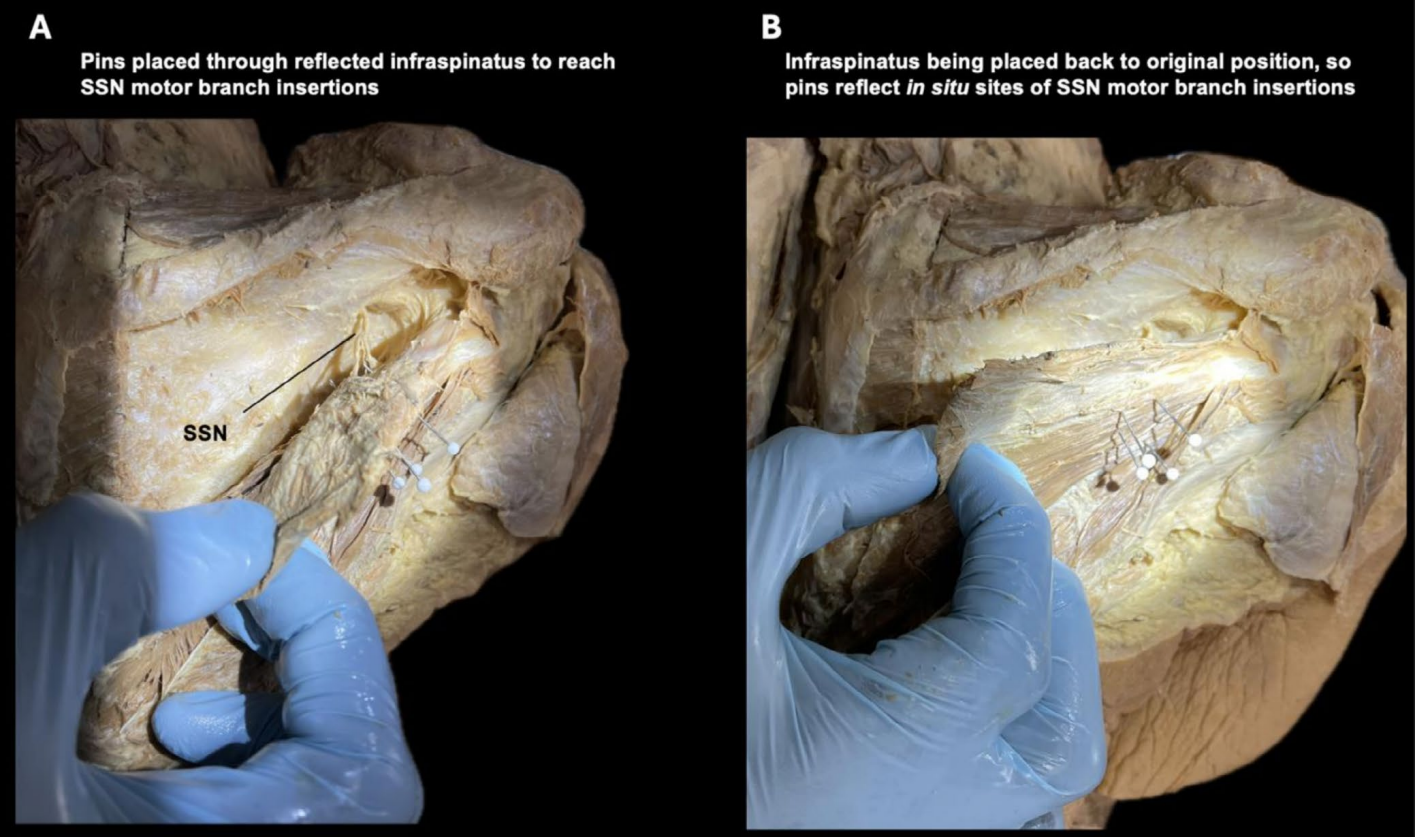
Identifying suprascapular motor branch insertions into infraspinatus (motor points). Defining sites of suprascapular nerve (SSN) motor branch entry into infraspinatus via reflection and pin placement.

### 2.5 Measuring Supraspinatus and Infraspinatus Motor Point Topography

Distances (mm) were measured along two axes for each of the muscles via ruler‐calibrated images and *ImageJ* software. Given the variable contours of musculature and the alterations with formalin fixation, a defined two dimensional plane was transposed over the supraspinatus and infraspinatus to inform distance measures; X (medial–lateral) and Z (anterior–posterior) distances from the average site of nerve entry into supraspinatus were measured from: (1) acromioclavicular joint (AC); (2) superior angle (SA); (3) root of the scapular spine (RS); (4) deltoid tubercle (DT); and (5) lateral acromion (LA; exemplified in Figure 4A).

**FIGURE 4:**
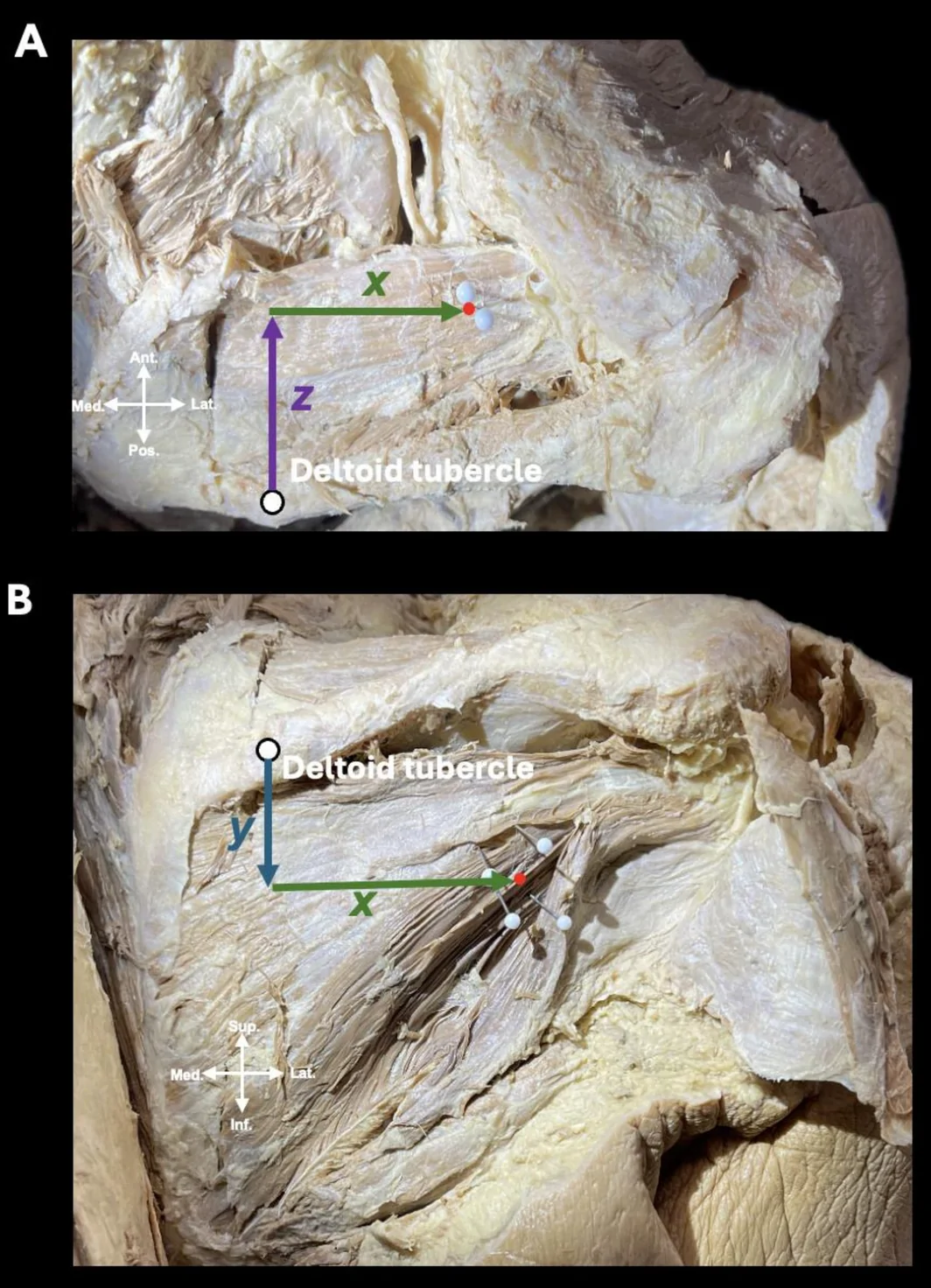
Measurement of distances from deltoid tubercle to average site of motor nerve entry into (A) supraspinatus and (B) infraspinatus. Distance measures in two axes from the deltoid tubercle to the injection target of (A) supraspinatus [superior view of right shoulder with trapezius reflected] and (B) infraspinatus [posterior view of the right shoulder with deltoid reflected]. In (A) X mm reflects the medial‐lateral distance and Z mm reflects the anterior–posterior distance. In (B) X mm reflects the medial‐lateral distance and Y mm reflects the superior–inferior distance. The red dot is equidistant from the pin placements that delineate sites of motor points; the rationale is that with a single injection localized near the sites of suprascapular nerve motor entry, sufficient coverage can be achieved, relieving myofascial pain.

X (medial‐lateral) and Y (superior–inferior) distances from landmarks to the average site of nerve entry into infraspinatus were measured from: (1) root of the scapular spine (RS); (2) inferior angle (IA); (3) deltoid tubercle (DT); and (4) lateral acromion (LA; exemplified in Figure 4B).

The specimens were placed in identical positions, guided by demarcations on a board, and camera placement was the same for all specimens, enabling consistency (Figure 5). The accuracy of image‐based measures was confirmed with in‐person digital caliper measures performed in triplicate by a single experimenter and performed blinded by a second experimenter.

**FIGURE 5:**
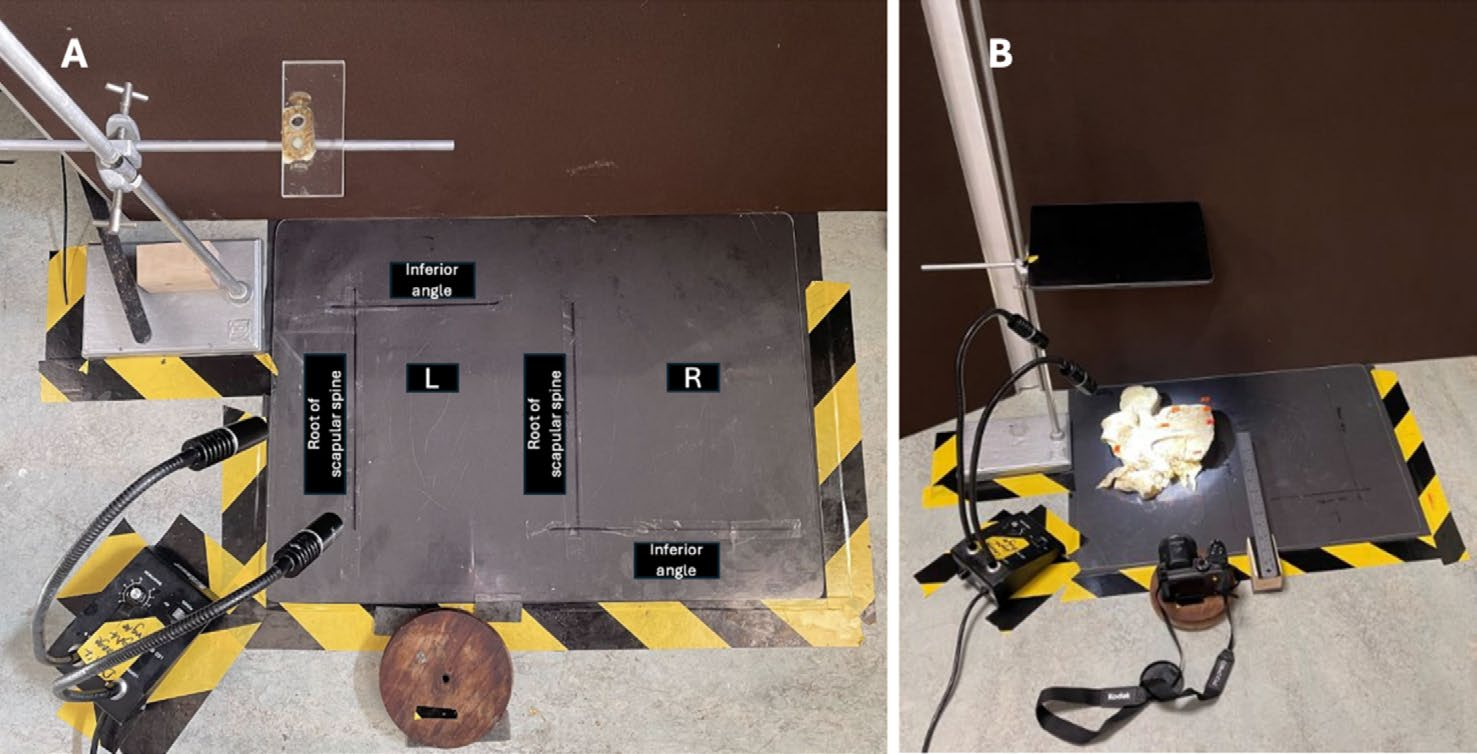
Experimental apparatus. L, left shoulder and R, right shoulder.

### 2.6 Statistical Analysis

Graphs were produced using Prism v10.4.2 (GraphPad). Statistical analyses were conducted using Prism and Microsoft Excel v16.95.4. Sample size is *n* = 20 throughout. The *n*‐value represents the number of shoulder specimens involved in the analyses. To test for normal distribution, the Shapiro–Wilk test (Shapiro and Wilk 1965) and Q–Q plots were used. Simple linear regression analysis was employed to test for correlation between variables, measured by *R* ^2^ and the Pearson correlation coefficient (*r*). The *F*‐test was used to investigate statistically significant, non‐zero relationships between variables. Statistical significance was attributed to differences for *p*‐values ≤ 0.05 (*p ≤ 0.05).

### 2.7 Dye Injection Validation

To validate the accuracy of landmark‐guided localization of motor points, injection via a 25‐gauge needle of 2.5 mL of 0.25% methylene blue dye solution was performed on four additional unpaired formalin‐fixed shoulder specimens (2 right and 2 left), with the skin attached. The specific approach to performing these validating injections was derived from the *Results*. Following injection, the specimens were dissected to assess for coverage of motor points.

The volume and needle size were selected to resemble ultrasound‐ and electromyography‐guided injections in clinical practice (Tan and Jia 2021; Yi et al. 2023); however, as no consensus guidelines for supraspinatus or infraspinatus have been reported to date, the volume of injectate and needle size were informed by other rotator cuff muscles, such as subscapularis.

## 3 Results

Measures of scapular dimensions (Table 1) and distances from landmarks to the average supraspinatus (Table 2) and infraspinatus (Table 3) motor points were performed on 20 shoulder specimens (10 pairs). A mean of 2.11 ± 0.32 (range: 2–3) motor points were identified in supraspinatus and 3.55 ± 0.62 (range: 2–5) in infraspinatus.

**TABLE 1:** Measurement of scapular dimensions.

	All specimens	Left side	Right side
Height	145.80 ± 15.19 (111.06–166.86)	147.61 ± 15.56	143.99 ± 15.42
Spine length	140.32 ± 14.31 (114.41–160.91)	137.87 ± 16.30	142.77 ± 12.37
Width	107.57 ± 12.43 (85.04–129.70)	106.58 ± 12.28	108.91 ± 13.13

*Note:* All measurements are in mm (mean ± SD). The range of measurements between the different shoulder specimens is indicated in brackets.

**TABLE 2:** X and Z distances from average location of supraspinatus motor points (SSNs) to scapular landmarks.

Relation	All specimens	Left side	Right side
X AC–SSNs	48.19 ± 13.14 (27.77–70.34)	50.89 ± 15.32	45.49 ± 10.67
Z AC–SSNs*	16.24 ± 11.71 (−4.74–33.59)	13.62 ± 12.32	18.86 ± 11.06
X SA–SSNs	57.09 ± 15.37 (36.14–97.08)	53.88 ± 11.73	60.31 ± 18.39
Z SA–SSNs	17.28 ± 10.1 (3.32–37.56)	20.56 ± 12.66	14.0 ± 5.6
X RS–SSNs	66.56 ± 16.2 (47.92–111.3)	65.14 ± 12.71	67.98 ± 19.7
Z RS–SSNs*	0.15 ± 8.2 (−17.53–14.0)	−1.16 ± 10.48	1.45 ± 5.33
X DT–SSNs	33.03 ± 12.21 (15.93–57.56)	30.67 ± 12.81	35.39 ± 11.77
Z DT–SSNs	20.69 ± 8.5 (2.49–32.31)	17.93 ± 8.88	23.45 ± 7.54
X LA–SSNs	69.19 ± 13.49 (48.21–91.74)	70.98 ± 16.26	67.39 ± 10.62
Z LA–SSNs	27.89 ± 10.93 (7.51–45.35)	23.08 ± 11.27	32.71 ± 8.57

*Note:* All values are in mm and mean ± SD (range). Note that * indicate two relations with negative values. Typically, average motor nerve entry into supraspinatus (SSNs) was anterior to the AC joint and root of the scapular spine (positive). In cases where these distances are negative, the average motor nerve entry into supraspinatus was posterior to the root of the scapular spine or AC joint. X indicates medial‐lateral distance and Z indicates anterior–posterior distance.

Abbreviations: X AC–SSNs, medial distance from the acromioclavicular joint to the average supraspinatus motor point; X DT–SSNs, lateral distance from the deltoid tubercle to the average supraspinatus motor point; X LA–SSNs, medial distance from the lateral acromion to the average supraspinatus motor point; X RS–SSNs, lateral distance from the root of the scapular spine to the average supraspinatus motor point; X SA–SSNs, lateral distance from the superior angle to the average supraspinatus motor point; Z AC–SSNs*, anterior distance from the acromioclavicular joint to the average supraspinatus motor point; Z DT–SSNs, anterior distance from the deltoid tubercle to the average supraspinatus motor point; Z LA–SSNs, anterior from the lateral acromion to the average supraspinatus motor point; Z RS–SSNs*, anterior distance from the root of the scapular spine to the average supraspinatus motor point; Z SA–SSNs, posterior distance from the superior angle to the average supraspinatus motor point.

**TABLE 3:** X and Y distances from average location of infraspinatus motor points (SSNi) to scapular landmarks.

	All specimens	Left side	Right side
X RS–SSNi	67.65 ± 11.09 (48.31–83.83)	65.61 ± 12.72	69.68 ± 9.41
Y RS–SSNi	21.38 ± 10.29 (2.94–42.06)	22.33 ± 7.66	20.44 ± 12.76
X IA–SSNi	49.99 ± 12.05 (28.43–76.31)	45.26 ± 9.51	54.71 ± 12.90
Y IA–SSNi	80.46 ± 10.69 (62.34–100.35)	81.30 ± 13.31	79.62 ± 7.92
X LA–SSNi	68.89 ± 10.27 (53.39–88.31)	68.84 ± 9.86	68.94 ± 11.20
Y LA–SSNi	48.51 ± 11.28 (29.89–76.99)	47.41 ± 14.01	49.62 ± 8.34
X DT–SSNi	37.13 ± 10.52 (19.45–60.43)	37.18 ± 12.82	37.08 ± 8.32
Y DT–SSNi	25.89 ± 8.82 (6.87–41.04)	27.67 ± 8.25	24.11 ± 9.43

*Note:* All values are in mm and mean ± SD (range). Summary table of the topographical relations of the average suprascapular nerve motor entry into infraspinatus (SSNi), the injection target, in the X (medial‐lateral) and Y (superior–inferior) axes.

Abbreviations: X DT–SSNi, Lateral distance from deltoid tubercle to average infraspinatus motor point; X IA–SSNi, Lateral distance from inferior angle to average infraspinatus motor point; X LA–SSNi, Medial distance from lateral acromion to average infraspinatus motor point; X RS–SSNi, Lateral distance from root of scapular spine to average infraspinatus motor point; Y DT–SSNi, Inferior distance from deltoid tubercle to average infraspinatus motor point; Y IA–SSNi, Superior distance from inferior angle to average infraspinatus motor point; Y LA–SSNi, Inferior distance from lateral acromion to average infraspinatus motor point; Y RS–SSNi, Inferior distance from root of scapular spine to average infraspinatus motor point.

There was variation in scapular size but no significant left vs. right differences (two‐tailed paired *t‐*test; *p* > 0.05).

There was variation in distances from landmarks to suprascapular nerve motor points, even between left and right shoulders for certain relations (Tables 2 and 3).

Simple linear regression assessed if there was a correlation between distance measures and scapular dimensions. Lateral distance from the superior angle to average supraspinatus motor point can be predicted by scapular height (*r* = 0.53; Figure 6A), spine length (*r* = 0.57; Figure 6B), or width (*r* = 0.52; Figure 6C). Lateral distance from the root of scapular spine to the average motor point may also be predicted by scapular height (*r* = 0.60; Figure 6A), spine length (*r* = 0.57; Figure 6B), or width (*r* = 0.51; Figure 6C). Based on spine length (*r* = 0.60; Figure 6B) or width (*r* = 0.66; Figure 6C), the anterior distance from the deltoid tubercle to the average motor point may be predicted. These findings indicate that the variable topography of supraspinatus motor points can be predicted using patient‐specific scapular dimensions.

**FIGURE 6:**
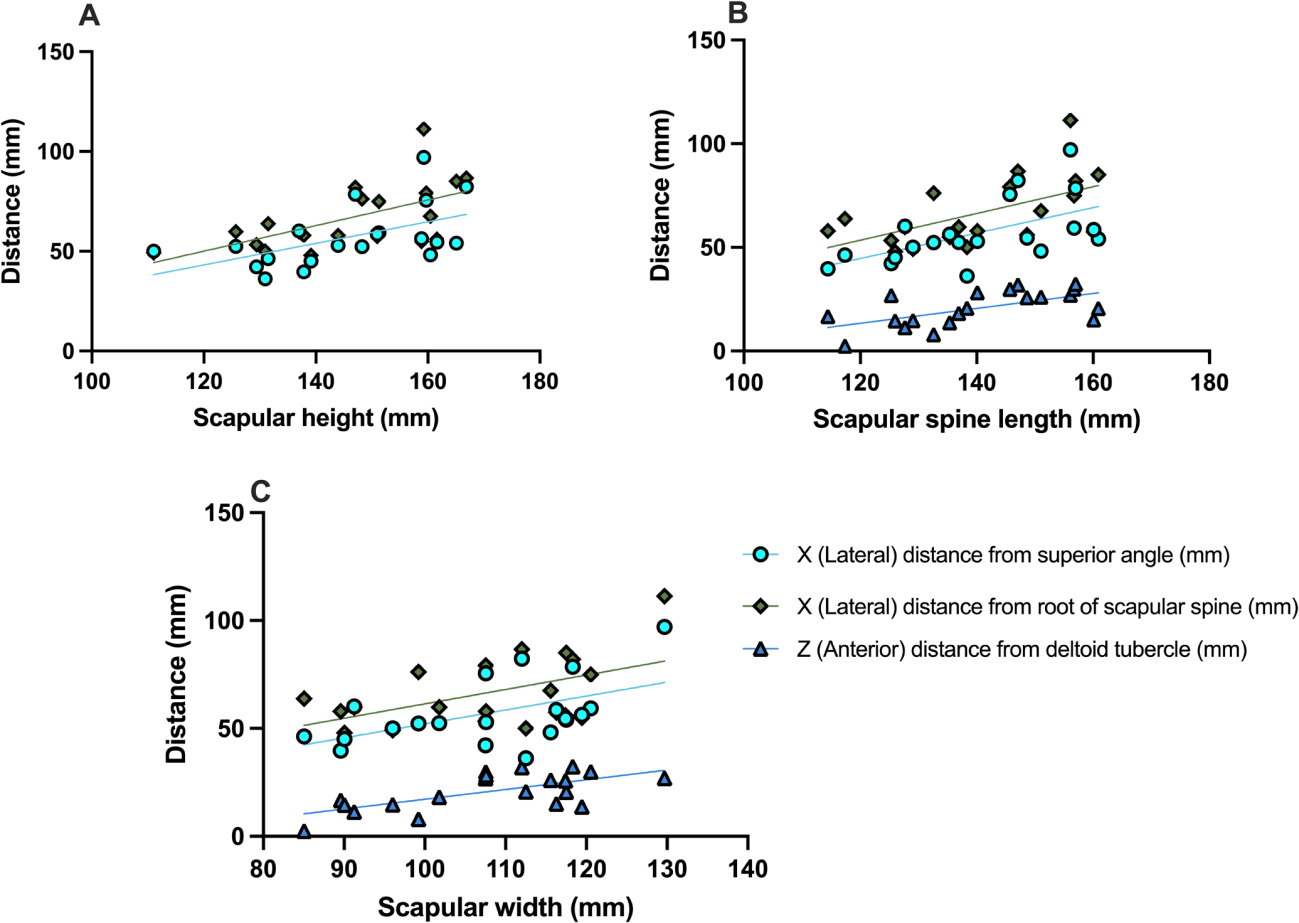
The topographical relations of supraspinatus motor points can be predicted by scapular dimensions. The topographical relations of supraspinatus motor points can be predicted by scapular dimensions. Scatter plot showing significant correlations between X and Z distances to the average motor nerve entry into supraspinatus and scapular (A) height, (B) spine length, and (C) width.

Based on scapular height, the lateral (*r* = 0.60; Figure 7A) and inferior (*r* = 0.46; Figure 7A) distances from the root of the scapular spine to the average infraspinatus motor point can be predicted. Based on scapular spine length or width, the medial (*r* = 0.44–0.58; Figures 7B,C) and inferior (*r* = 0.50–0.58; Figures 7B,C) distances from the lateral acromion to the average infraspinatus motor point can be predicted. This suggests that the topography of infraspinatus motor points can be predicted by scapular dimensions.

**FIGURE 7:**
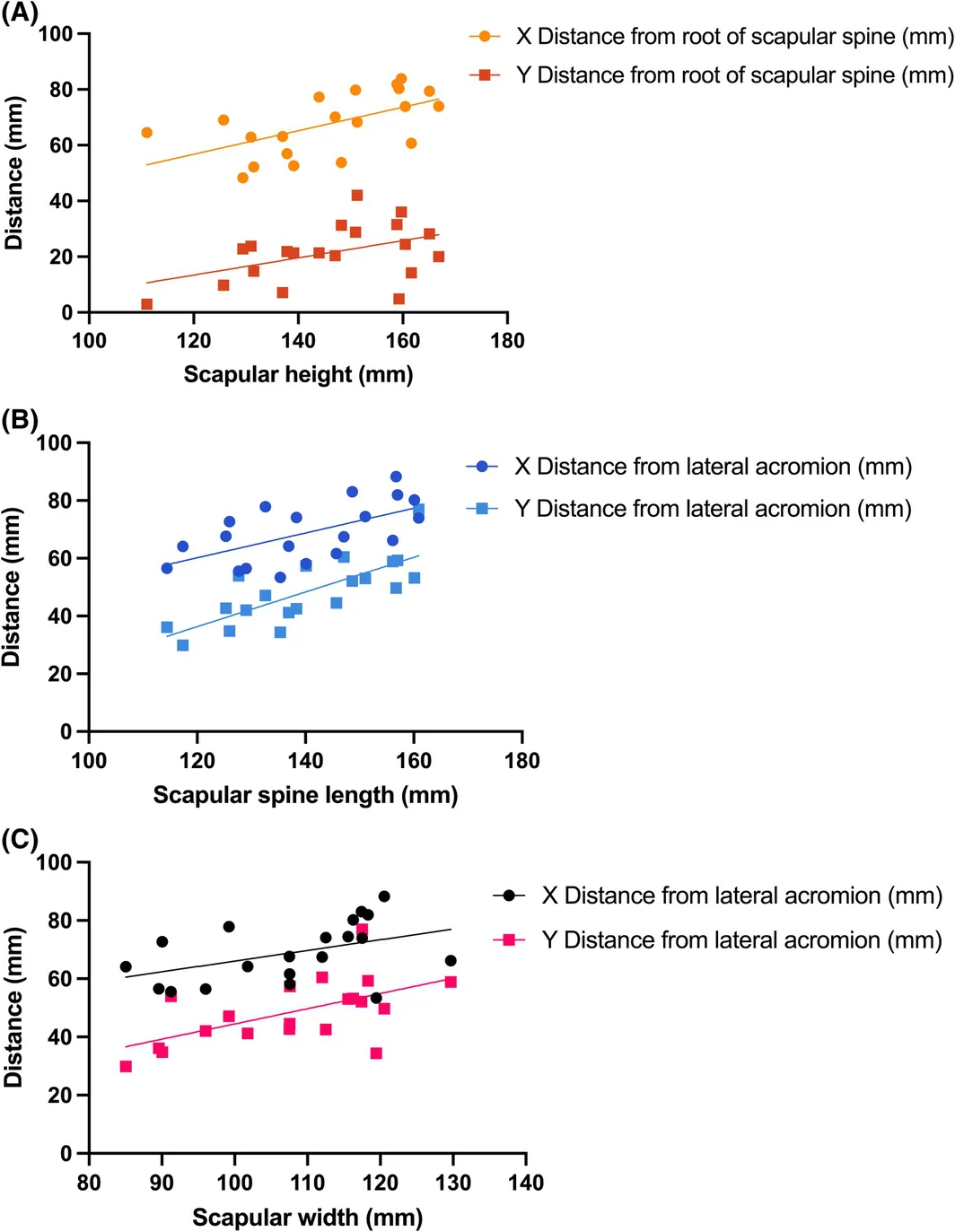
The topographical relations of infraspinatus motor points can be predicted by scapular dimensions. For the lateral acromion, X distance is medial, and Y is inferior. For the root of the scapular spine, X distance is lateral, and Y is inferior.

This suggests that for certain landmarks, patient‐specific scapular dimensions can be used to inform injection approaches to target supraspinatus and infraspinatus motor points. This notion was anatomically validated via blind methylene blue dye injections into four additional shoulder specimens, distinct from the original sample.

## 4 Development and Preclinical Validation of a Landmark‐Guided Injection Protocol

To simulate clinically translatable, landmark‐guided injections, the following procedural steps were undertaken to validate the accuracy of targeting supraspinatus and infraspinatus motor points:

1. *Measurement of Scapular Dimensions*

Each shoulder specimen, with intact skin overlying the supraspinatus and infraspinatus, was measured for scapular height, spine length, and width. These measures were used to personalize the injection approaches. All three scapular dimensions yielded near identical predicted distances for injection targeting. Scapular spine length was selected for presentation due to its simplicity and ease of measurement.

2 *Application of Patient‐Specific Predictive Models*

The recorded scapular spine lengths were inputted into linear regression equations derived from dissected specimens (see *Results*, Tables 4 and 5) to generate predicted coordinates for the average site of supraspinatus motor points.

- For supraspinatus injection, two distances were calculated:
  ○ X‐distance from the root of the scapular spine (medial–lateral)
    - [equation: X‐distance = 0.3595*(spine length) − 29.76]
  ○ Z‐distance from the deltoid tubercle (anterior–posterior)
    - [equation: Z‐distance = 0.6444*(spine length) − 23.85]

**TABLE 4:** Linear regression line of best fit equations, *R* ^2^, and *p*‐values from plots in Figure 6.

Graph	Distance (X/Y) from landmark to injection site vs. scapular dimension	Line of best fit equation	*R* ^2^	*p*
A	X SA–SSNs vs. height	Y = 0.5409*X—21.78	0.2858	0.0152
	X RS–SSNs vs. height	Y = 0.6402*X—26.77	0.3603	0.0051
B	X SA–SSNs vs. spine length	Y = 0.6131*X—28.93	0.3256	0.0086
	X RS–SSNs vs. spine length	Y = 0.6444*X—23.85	0.3238	0.0088
	Z DT–SSNs vs. spine length	Y = 0.3595*X—29.76	0.3660	0.0047
C	X SA–SSNs vs. width	Y = 0.6460*X—12.51	0.2729	0.0181
	X RS–SSNs vs. width	Y = 0.6672*X—5.320	0.2621	0.0210
	Z DT–SSNs vs. width	Y = 0.4511*X—27.91	0.4350	0.0016

*Note:* Summary statistics of simple linear regression analyzing the relationship between the average motor nerve entry into supraspinatus (SSNs) and scapular dimensions.

Abbreviations: X RS–SSNs, lateral distance from the root of the scapular spine to the average supraspinatus motor point; X SA–SSNs, lateral distance from the superior angle to the average supraspinatus motor point; Z DT–SSNs, anterior distance from the deltoid tubercle to the average supraspinatus motor point.

**TABLE 5:** Linear regression line of best fit equations, *R* ^2^, and *p*‐values from plots in Figure 7.

Graph	Distance (X/Y) from landmark to injection site vs. scapular dimension	Line of best fit equation	*R* ^2^	*p*
A	X RS–SSNi vs. height	Y = 0.4290*X + 8.698	0.3570	0.0054
	Y RS–SSNi vs. height	Y = 0.3089*X—23.65	0.2080	0.0433
B	X LA–SSNi vs. spine length	Y = 0.3666*X + 29.39	0.1968	0.0500
	Y LA–SSNi vs. spine length	Y = 0.5230*X—7.837	0.3324	0.0078
C	X LA–SSNi vs. width	Y = 0.4223*X + 6.077	0.3345	0.0076
	Y LA–SSNi vs. width	Y = 0.3555*X—30.75	0.2450	0.0265

*Note:* Summary statistics of simple linear regression analyzing the relationship between the average motor nerve entry into infraspinatus (SSNi) and scapular dimensions.

Abbreviations: X LA–SSNi, medial distance from lateral acromion to average infraspinatus motor point; X RS–SSNi, lateral distance from root of scapular spine to average infraspinatus motor point; Y LA–SSNi, inferior distance from lateral acromion to average infraspinatus motor point; Y RS–SSNi, inferior distance from root of scapular spine to average infraspinatus motor point.

These values defined the relative projected coordinate of the average supraspinatus motor point (injection target) deep to the skin surface and trapezius (Figure 8A).

- For infraspinatus, lateral acromion was used as a single reference point. The root of the scapular spine could also have been used, but the authors found that lateral acromion was more easily palpable in specimens of variable adiposity or muscularity. The corresponding X (medial‐lateral) and Y (superior–inferior) distances were calculated based on scapular spine length to localize the predicted average motor point for injection:
  ○ X (medial‐lateral) and Y (superior–inferior) distances from the lateral acromion
    - [equations: X‐distance = 0.3666*(spine length) + 29.39; Y‐distance = 0.5230*(spine length) − 7.837].

These values defined the relative projected coordinate of the average infraspinatus motor point (injection target) deep to the skin surface and deltoid (Figure 8B).

**FIGURE 8:**
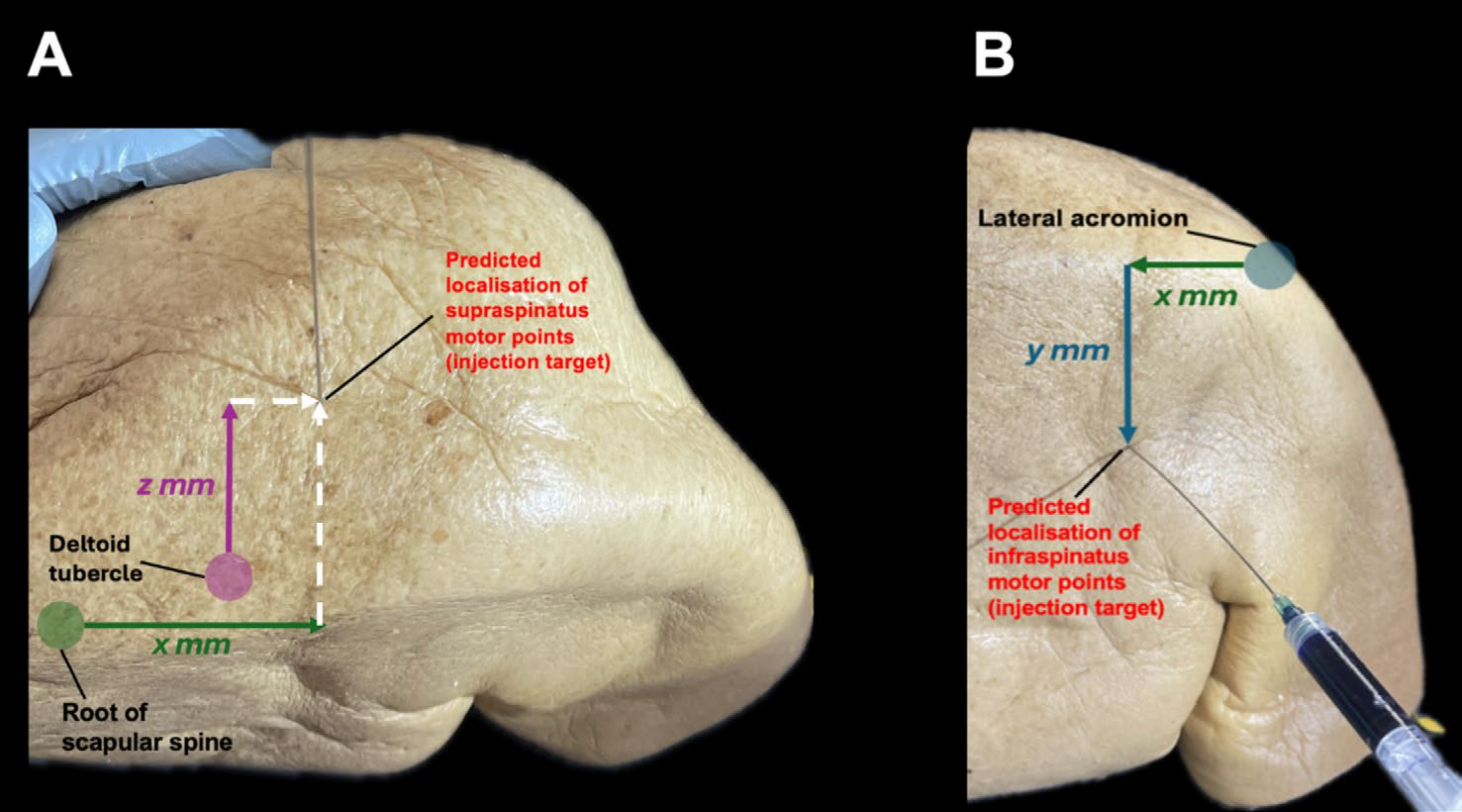
Landmark‐guided approaches for injecting supraspinatus and infraspinatus. (A) superior view of right shoulder demonstrating the injection approach for supraspinatus. (B) posterior view of right shoulder demonstrating the injection approach for infraspinatus.

3 *Landmark‐Guided Injection Technique*

The defined injection sites were marked on the skin surface using the calculated distances from the relevant palpated bony landmarks. A 25‐gauge needle was inserted perpendicular to the skin at the predicted coordinate and advanced until resistance indicated contact with the underlying bony floor of the supraspinous or infraspinous fossa, simulating clinical depth. A volume of 2.5 mL of 0.25% methylene blue dye was injected to simulate BoNT delivery.

This image‐free, patient‐specific protocol enabled consistent and anatomically accurate targeting of supraspinatus and infraspinatus motor points, as validated through post‐injection dissection. All four specimens demonstrated complete coverage of both supraspinatus and infraspinatus motor points (Figure 9).

**FIGURE 9:**
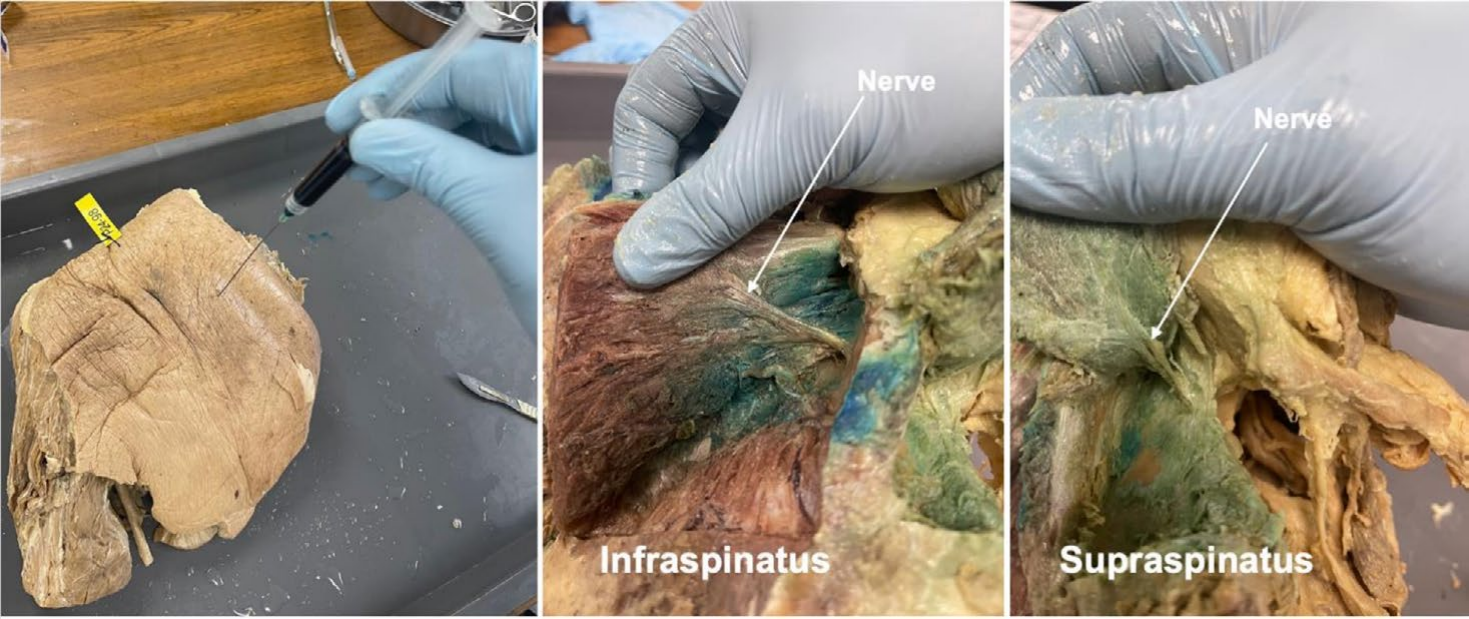
Dye injection validation on a left shoulder specimen showing coverage of both infraspinatus and supraspinatus motor points. Example of a left shoulder specimen injected by methylene blue dye to target supraspinatus and infraspinatus motor points. Figure shows injection of infraspinatus, sufficient dye coverage of motor points of both infraspinatus and supraspinatus based on patient‐specific, landmark‐guided injection.

## 5 Discussion

This cadaveric study demonstrates that patient‐specific scapular dimensions can predict the topographical location of supraspinatus and infraspinatus motor points. Harnessing this finding, we propose a novel landmark‐guided approach for botulinum neurotoxin (BoNT) injection into these muscles. In this approach, the needle is injected a defined distance from palpable scapular landmarks to reach the predicted motor points. These distances are patient‐specific, informed by scapular size, and easily measurable in clinic. This approach enables image‐free targeting of motor points and was validated via successful dye injection in all tested specimens.

Anatomical studies have suggested variability in the topography of supraspinatus and infraspinatus motor points (Lee et al. 2022, 2023). Through mapping these motor points, these studies developed ultrasound‐guided injection techniques for BoNT delivery. Alongside electromyographic guidance, ultrasound‐guided BoNT injections are imperative in treating myofascial pain (Evans and Porter 2015; Tan and Jia 2021). However, such modalities are not universally available, particularly in low‐resource settings (Becker et al. 2016). Another approach to BoNT delivery for myofascial pain involves tender point palpation and blind injection where the skeletal muscle band is most tender and taut (Climent et al. 2013). However, this technique has shown limited long‐term success in treating pain (Hollingworth et al. 1983; Rubin et al. 2009).

Our study establishes reproducible, landmark‐guided approaches that do not require specialist equipment, are anatomically validated, and are patient‐specific. The technique relies on measurements in two anatomical axes relative to palpable landmarks. This confers directional specificity that enables clinicians to determine where to inject in relation to scapular landmarks.

These findings have important implications for improving the precision of BoNT injections. The therapeutic effect of BoNT is dependent on its proximity to motor endplates, approximately where motor nerves enter muscle (Harrison et al. 2007; Miguel and Cirera 2021). By localizing these motor points via palpable scapular landmarks, our method may allow more accessible and standardized delivery of BoNT injections.

Numerous scapular landmarks showed utility in guiding intramuscular BoNT injections into the rotator cuff, such as the root of the scapular spine, deltoid tubercle, and lateral acromion. In particular, the deltoid tubercle represents a landmark not previously exploited or appreciated in injection planning.

Validation using 2.5 mL methylene blue injections confirmed the accuracy of our proposed landmark‐guided injections. This injectate volume is comparable to clinical volumes, suggesting high translational potential. Further enhanced motor point targeting could reduce required dosages, lower costs, and minimize off‐target effects, such as unwanted paralysis (Carré et al. 2024). The landmark‐guided approaches may also be relevant for intramuscular lidocaine or corticosteroid injections in managing rotator cuff pain of myofascial or neurogenic (e.g., hemiplegic shoulder) origin (Anwar et al. 2024).

Via the findings from this study, we proposed a protocol for performing BoNT injections, guided by patient‐specific anatomy and scapular landmarks. Scapular spine length provides a practical reference for personalizing injections, though height and width offer comparable utility. All are readily measurable via surface palpation.

Nevertheless, the findings yield important clinical considerations:

- Scapular dimensions allow for patient‐specific adjustments, supporting personalized medicine.
- This technique may be useful in settings lacking ultrasound or electromyography.
- Injection depth (not informed by the present study) should be adjusted based on individual patient anatomy, accounting for variation in subcutaneous fat, muscle bulk, and scapular contour.
  ○ In patients with greater muscle bulk, a deeper insertion may be required.
  ○ In frail or elderly patients, inject more superficially.
- With the growing adoption of automation in medicine, the study's linear equations could support future development of algorithmic injection guides or point‐of‐care tools.

## 6 Limitations and Future Directions

Despite the strengths of this study, several limitations should be acknowledged. The use of elderly, formalin‐fixed cadaveric specimens may not reflect the tissue characteristics of younger patients in vivo*;* fresh frozen specimens are thought to better resemble the living state and may have offered greater utility (Jansen et al. 2020). However, muscle atrophy and post‐fixation shrinkage, coupled with the lack of blood flow, may still have altered the spread of injectate compared to the in vivo state (Tran et al. 2015). Furthermore, just 10 unique individuals (20 shoulders) were included to generate the injection approaches, indicative of a small sample size. Similarly, the restricted dye validation sample (*n* = 4) is a limitation, even though all demonstrated accurate coverage. In addition, we did not include a dry bone validation cohort. Such an approach–or comparable radiological validation using CT –could strengthen reproducibility testing by confirming the reliability of scapular landmarks across larger, more diverse populations. This is an avenue for future investigation. Moreover, while we correlated sites of anatomical motor points with scapular dimensions, we could not confirm functional motor endplate activity with electromyography. In the future, this scapular dimension‐informed, landmark‐guided localization of suprascapular nerve motor endplates could be confirmed in living patients via both electromyography and ultrasound.

This anatomical framework for patient‐specific injections could be extended beyond the rotator cuff to other muscles with defined motor points. Moreover, some studies suggest multiple injections, rather than a single injection, into infraspinatus may improve outcomes (Lim et al. 2008); therefore, a dual‐point injection strategy, informed by landmarks, could be explored.

## 7 Conclusion

This study presents a pre‐clinically validated, patient‐specific, landmark‐guided approach for intramuscular BoNT injection into the rotator cuff. By leveraging predictable correlations between scapular dimensions and the topography of supraspinatus and infraspinatus motor points, this approach enables accurate, image‐free injection targeting with broad clinical utility. These findings represent a translational step toward more accessible and precise treatment of shoulder pain.

## Funding

This work was funded in part by the Royal College of Surgeons of England Intercalated Bachelor of Science Degree in Surgery Award.

## Ethics Statement

The donors had provided consent for anatomical research prior to decease in compliance with the Human Tissue Act 2004.

## Data Availability

The data that support the findings of this study are available from the corresponding author upon reasonable request.
